# Sexual quality of life assessment in young women with breast cancer during adjuvant endocrine therapy and patient-reported supportive measures

**DOI:** 10.1007/s00520-022-06810-3

**Published:** 2022-01-14

**Authors:** Angelique Bobrie, Marta Jarlier, Aurore Moussion, William Jacot, Veronique D’Hondt

**Affiliations:** 1 Medical Oncology Department, Institut du Cancer de Montpellier (ICM), 208 Avenue des Apothicaires, 34298 Montpellier Cedex5, France; 2 Institut de Recherche en Cancerologie de Montpellier (IRCM), INSERM, University of Montpellier, Montpellier, France; 3 Biometrics Unit, Institut du Cancer de Montpellier, Montpellier, France; 4 Medical Research Unit, Institut du Cancer de Montpellier, Montpellier, France

**Keywords:** Sexual quality of life, Patient-reported measures, Breast cancer, Supportive care, Survivorship

## Abstract

**Purpose:**

Sexual quality of life (QoL) is affected during and after breast cancer (BC) treatment. The purpose was to investigate sexual and global QoL and patient-reported measures to address this issue in young women (< 51 years) with BC after the acute treatment phase, during adjuvant endocrine therapy.

**Methods:**

Three EORTC questionnaires and an additional specific questionnaire, developed for the study, were used to assess sexual and global QoL and patient-reported supportive measures in BC patients who had received their endocrine therapy for at least 24 months. Among the 54 eligible patients, 45 (83%) agreed to participate in the study.

**Results:**

We showed a deterioration in sexual QoL and poor communication with healthcare professionals. Most patients (88.9%) declared that it was important that sexuality should be discussed with caregivers and that the partner should also be involved. Most patients (60%) had taken at least one action to overcome their sexual problems. Most of these interventions (63%) originated from the patient herself.

**Conclusions:**

Sexual QoL is a major issue in young BC patients and is poorly addressed by healthcare professionals. Most of the supportive methods used by the patients to overcome these side effects were on their own initiative. Communication and counseling on sexuality by healthcare professionals need to be improved during BC treatment. Patients suggested supportive measures they would find useful and appropriate to develop in the clinic. The final goal is to improve the sexual QoL of BC patients with the appropriate intervention and support.

**Supplementary Information:**

The online version contains supplementary material available at 10.1007/s00520-022-06810-3.

## Introduction

Advances in BC diagnosis and treatment have led to the improvement of the patients’ prognosis, with a 5-year survival in wealthier countries now reaching 90% [1]. The disease and its treatment can affect the day-to-day lives of BC survivors, and these physical, psychological, and social concerns become important issues to be addressed in clinic [2]. Among these QoL concerns, sexual difficulties have been more recently studied [3–7]. In this regard, a specific health-related quality of life questionnaire (HRQLQ) dedicated to evaluating sexual dysfunction related to the disease and its treatment, in cancer patients, the EORTC SHQ-22, has recently been developed [8].

We previously published the results of a longitudinal assessment analyzing the sexual and global QoL of 106 women with hormone receptor-positive (HR +) BC during the first year of endocrine therapy, using the EORTC SHQ-22 and global HRQLQ [9]. This study showed a deterioration of patients’ sexual QoL as compared to women in the general population and a very low communication level about sexual themes between patients and healthcare givers.

In this new study, we focused on young patients less than 51 years old, a population whose sexual QoL is more frequently and severely affected following BC care [9–13]. We analyzed the sexual and global QoL in women with HR + BC who had received their endocrine therapy for at least 24 months, to analyze sexual dysfunction long after the acute surgical, chemotherapeutic, and radiotherapeutic treatment phases. We also used an additional specific questionnaire, proposed in the context of our study, to question women on the actions taken individually to address sexual issues and which supportive measures they would find useful and appropriate to develop in the clinic.

## Materials and methods

### Study design and subject recruitment

This unicentric prospective study (CUPIDON 2) was conducted at the Institut du Cancer de Montpellier (ICM). The protocol was approved by the French Ethics Committee and the internal review board of the institution. Patients received an information letter and all provided written consent before enrolment. The primary objective was to evaluate the sexual QoL of women younger than 51 years old with HR + BC who had received their endocrine therapy for at least 24 months using the EORTC SHQ-22 questionnaire. The secondary objectives were: (1) to evaluate the global QoL at the same time-point using the EORTC QLQ-C30 [14] and QLQ-BR23 [15] questionnaires; (2) to evaluate the information received by patients about sexual issues; (3) to evaluate the need for specific management of sexual troubles; (4) to describe supportive measures or therapeutic interventions used by women on their own initiative using the specific Cupidon questionnaire, proposed for the study. Eligible women were between 18 and 51 years old, self-declared sexually active, displaying HR + early BC, and having completed surgery as well as chemotherapy and/or radiotherapy when indicated. They all had received their endocrine therapy for at least 24 months.

### Data and measures

General health and socio-demographic information, including employment status, physical activity, and partner status, were self-reported by participants. Age, weight, size, menopausal status, pathology report, type of treatment (type of surgery, radiotherapy, and chemotherapy) as well as comorbidities were collected from the medical records.

Patients were invited to complete the three EORTC HRQLQ only once.

The general EORTC QLQ-C30 questionnaire dedicated to all cancer patients includes 30 items assessing the global health status with 5 functional scores (physical, role, cognitive, social, and emotional) and 9 symptom scores (nausea and vomiting, pain, fatigue, dyspnea, sleep disturbances, appetite loss, constipation, diarrhea, and financial difficulties).

The specific EORTC QLQ-BR23 module dedicated to breast cancer patients includes 23 items allowing to assess 4 functional scores (body image, sexual functioning, sexual enjoyment, and future perspective) and 4 symptom scores (systemic side effects, arm symptoms, breast symptoms, and being upset by hair loss).

The EORTC SHQ-22 is a multi-dimensional QoL instrument used to measure sexual health in patients with cancer (men or women). This new tool covers both sexual functioning and psychosexual components. It includes 8 items on sexual satisfaction, 3 items on sexual pain, and 11 single items in an integrative approach, leading to 7 functional scales and 4 symptom scales.

In these questionnaires, higher scores in the functioning scales indicate a better functional level, whereas higher scores in the symptom scales indicate the severity of problems. A user agreement for the use of the EORTC SHQ-22 was signed.

In addition, a homemade questionnaire containing 11 questions was proposed to patients in order to explore (1) the information regarding sexuality received during treatment, (2) the supportive measures or therapeutic interventions used by patients to overcome sexual dysfunction, and (3) the need and acceptability for specific management of sexual dysfunction. This specific Cupidon questionnaire is reproduced in extenso in the supplemental data.

### Statistical considerations

In this descriptive study, the sample size was based on the recruitment capacity of our institution, which represented a potential of around 50 patients meeting the inclusion criteria of the study for the planned duration. Considering an 80% acceptance rate (20% refusal), a total of 40 patients were expected to participate. The inclusion of at least 40 patients permits to estimate (for descriptive purposes) the mean scores of the dimensions of the EORTC SHQ-22 (ranging from 0 to 100) and its 95% confidence intervals (95% CI) with a width of 15 and hypothesizing a standard deviation of 24 [16].

Scorings of the three EORTC HRQLQ were calculated according to the EORTC Scoring Manuals, and results were presented as means and standard deviations (SD). Categorical variables were described using frequencies and percentages.

Statistical analysis was performed using the Stata v16 software (College Station, TX, 2019, USA).

## Results

### Patients’ characteristics

Among the 65 patients screened during consultations between February and April 2020, to participate in the study, 11 were not eligible (8 had no sexual activity, 2 had relapsed, 1 had discontinued her endocrine therapy), and 9 refused to participate. A total of 45 patients have been included and completed the four questionnaires. Clinical and socio-demographic characteristics are summarized in Table 1. The median age was 45 years. A majority of women had been treated by breast conserving surgery (62%), and had received chemotherapy (87%). Most women (87%) had received endocrine treatment for 2 to 5 years. Sixty percent of women were premenopausal at cancer diagnosis. Endocrine therapy consisted mostly of tamoxifen (73%), and included a chemical castration in 18% of cases. According to the body mass index (BMI), 33 and 11% of patients were overweight or obese, respectively. Most patients (58%) practiced regular physical activity, i.e., minimum of 30 min, 3 times a week, and 76% were professionally active. All women were sexually active at baseline (inclusion criteria) and 17% declared a previous history of sexual problems either in the couple (5%) or of their partner (12%).

**Table 1:** Clinical and socio-demographic characteristics of the women

	Total (*n* = 45)
Median age–years (range)	45 (33–50)
Surgery	
Breast conserving	28 (62%)
Mastectomy	17 (38%)
Adjuvant chemotherapy	
Yes	39 (87%)
No	6 (13%)
Adjuvant radiotherapy	
Yes	41 (91%)
No	4 (9%)
Adjuvant endocrine therapy	
Tamoxifen	33 (73%)
Tamoxifen followed by LH-RH agonist + AI	5 (11%)
Tamoxifen followed by AI	4 (9%)
LH-RH agonist + AI	3 (7%)
Time since the beginning of the endocrine therapy	
2–5 years	39 (87%)
> 5 years	6 (13%)
Menopausal status at cancer diagnosis	
Premenopausal	27 (60%)
Postmenopausal	18 (40%)
Body mass index	
Median	23.6
< 18	3 (7%)
18–24	22 (49%)
25–29	15 (33%)
≥ 30	5 (11%)
Children	
Yes	38 (84%)
No	7 (16%)
Physical activity	
Yes	26 (58%)
No	19 (42%)
Smoker	
Yes	14 (31%)
No	16 (36%)
Stopped	15 (33%)
Professional activity	
Worker	34 (76%)
Unemployed	10 (22%)
Retired	1 (2%)
History of sexual problem	
Within the current couple	2 (5%)
Of the partner	5 (12%)

Abbreviations: *AI*, aromatase inhibitor; *LH-RH*, luteinizing hormone-releasing hormone

### Sexual health questionnaire

Mean scores for the EORTC SHQ-22 questionnaire are shown in Table 2. The importance attributed to sexual activity appeared relatively preserved (mean score of 60.00), but sexual satisfaction was lower (mean score of 45.80). The libido and the impact of treatment on sexual life scores were low (mean scores of 29.63 and 37.78) and the communication about sexuality with professionals score was extremely low (mean score of 11.11). The feeling of security with the partner and the femininity were less altered (mean scores of 52.71 and 59.69 respectively).

**Table 2:** Sexual quality of life assessed by the EORTC SHQ-22

EORTC SHQ-C22	*N* = 45		
	*N*	Mean	SD
Functional scales			
Sexual satisfaction	45	45.80	22.89
Importance of sexual activity	45	60.00	34.52
Libido	45	29.63	33.50
Impact of treatment on sexual life	45	37.78	39.31
Communication with professionals	45	11.11	23.57
Security with partner	43	52.71	40.00
Femininity	43	59.69	42.14
Symptom scales			
Sexual pain	41	31.98	32.03
Worrying about incontinence	44	9.09	24.23
Fatigue	44	49.24	39.69
Vaginal dryness	37	57.66	42.05

*N*, number of women who answered the question. Higher scores in the functioning scales indicate better functional level whereas, higher scores in the symptom scales indicate the severity of the symptoms

Vaginal dryness was the most important symptom (a mean score of 57.66). Fatigue appeared important (mean score of 49.24) as well as pain related to intercourses (a mean score of 31.98). Worrying about incontinence was rare (mean score 9.09).

### Quality of life questionnaires

Mean scores for the EORTC QLQ-C30 and the EORTC QLQ-BR23 are shown in Table 3.

**Table 3:** Global quality of life assessed by the EORTC QLQ-C30 and the EORTC QLQ-BR23

	*N* = 45		
	N	Mean	SD
EORTC QLQ-C30			
Functional scales			
Global health status	45	69.63	17.60
Physical functioning	45	87.85	12.89
Role functioning	45	82.22	24.46
Emotional functioning	45	55.37	32.18
Cognitive functioning	45	59.26	31.89
Social functioning	45	80.37	23.65
Symptom scales			
Fatigue	45	41.48	27.67
Nausea and vomiting	45	4.07	9.51
Pain	45	27.04	29.15
Dyspnea	45	22.96	24.44
Insomnia	45	46.67	43.46
Appetite loss	45	7.41	21.19
Constipation	45	11.85	22.65
Diarrhea	45	8.89	16.51
Financial difficulties	45	13.33	31.30
EORTC QLQ-BR23			
Functional scales			
Body image	45	66.30	30.25
Sexual functioning	45	35.19	24.68
Sexual enjoyment	34	62.75	28.15
Future Perspective	45	40.74	31.69
Symptom scales			
Systemic therapy side effects	45	26.56	17.69
Breast symptoms	44	22.73	18.97
Arm symptoms	44	22.73	23.96
Upset by hair loss	0		

*N*, number of women who answered the question

According to the EORTC QLQ-C30, the global health status score was 69.63. The mean scores of physical functioning, role functioning, and social functioning appeared better than those of emotional and cognitive functioning.

Regarding the symptom scales, the highest scores were fatigue and insomnia (mean scores of 41.48 and 46.67, respectively).

According to the EORTC QLQ-BR23, the score for the body image was 66.30, while the score for the sexual functioning scale appeared low (a mean score of 35.19). Sexual enjoyment appeared better (a mean score of 62.75), but only 75% of patients answered this item. The side effects of systemic therapy and local breast and arm symptoms had similar scores. With a minimum of 2 years since the end of the chemotherapy, women were no more concerned by hair loss.

### Specific Cupidon questionnaire

The answers to the specific Cupidon questionnaire are shown in Table 4.

**Table 4:** Patient-reported communication with health professionals and supportive measures and patients’ suggestions for sexual care, assessed by the CUPIDON questionnaire

Evaluation of the received information and satisfaction	*N* = 45	%
1. Did you receive any information from the medical oncologist or another caregiver on the possible impacts of cancer and of your treatment on sexuality?		
No	24	53.3
Yes^1^	12	26.7
I don’t remember	9	20.0
^1^Among the 12 patients: 10 were satisfied (agreed/fully agreed) by the received information and 2 had no opinion (question 2)		
2. I’m satisfied with the information I received about the possible impacts of cancer and the treatments on my sexuality		
Strongly disagree/disagree	14	31.1
No opinion	19	42.2
Agree/fully agree	12	26.7
3. Have you had any discussion with the oncologist or another caregiver from the Cancer Center about potential sexual problems?		
No	39	86.7
Yes	6	13.3
**Talking about sexual health and barriers**	*N* = 39	%
4. I did not discuss about sexuality because:		
I had no question or I didn’t feel the need to discuss about it		
Strongly disagree/disagree	6	15.4
No opinion	6	15.4
Agree/fully agree	27	69.2
I considered sexuality as a minor issue compared to cancer treatment		
Strongly disagree/disagree	9	23.1
No opinion	4	10.2
Agree/fully agree	26	66.7
Physicians and/or nurses looked in a hurry or too busy		
Strongly disagree/disagree	27	69.2
No opinion	2	5.1
Agree/fully agree	10	25.7
I felt too shy and/or discomfort to discuss this topic		
Strongly disagree/disagree	23	59.0
No opinion	4	10.2
Agree/fully agree	12	30.8
I felt discomfort since my physician was a man and/or since I had no positive contact with him/her		
Strongly disagree/disagree	34	87.2
No opinion	1	2.6
Agree/fully agree	4	10.2
It is in contradiction with my education, beliefs, culture		
Strongly disagree/disagree	36	92.3
No opinion	0	0.0
Agree/fully agree	3	7.7
**Management of sexual issues: evaluation of the needs in information and therapeutic proposals**	*N* = 45	%
5. In my opinion, it is important that a physician and/or a caregiver from the Cancer Center addresses the issue of sexuality and its potential dysfunction due to the cancer or to cancer treatments		
Strongly disagree/disagree	1	2.2
No opinion	4	8.9
Agree/fully agree	40	88.9
6. In my opinion, it is important that a physician and/or a caregiver from the Cancer Center addresses the issue of sexuality and its potential dysfunction due to the cancer or to cancer treatments with my partner		
Strongly disagree/disagree	5	11.1
No opinion	6	13.3
Agree/fully agree	33	73.4
No partner	1	2.2
7. I would have wished that a consultation about sexuality was systematically proposed at the beginning of cancer treatment		
Strongly disagree/disagree	12	26.7
No opinion	9	20.0
Agree/fully agree	24	53.3
8. I would have wished that a consultation about sexuality was available in the Cancer Center when needed during my treatment		
Strongly disagree/disagree	2	4.4
No opinion	9	20.0
Agree/fully agree	34	75.6
9. Have you been using any of these methods for sexual purpose since the beginning of your disease? (each sub-item was binary, only category ‘ yes’ is shown)		
Consultation with a psychologist	8	17.8
Consultation with a psychiatrist	2	4.4
Consultation with a sexologist	1	2.2
Pelvic floor physical therapy	3	6.7
Use of vaginal moisturizer	22	48.9
Use of vaginal laser therapy	0	0.0
Use of at least one the methods mentioned above	27	60.0
	*N* = 27	%
10. If you have been using any method from the previous question, have you decided it on your own?		
Yes (own initiative)	17	63.0
Yes (own initiative) and advice (from my: oncologist (*n* = 1), my gynecologist (n = 1))	2	7.4
No: advice^2^	8	29.6
^2^ from: oncologist (*n* = 1), general practitioner (*n* = 2), gynecologist (*n* = 5)		
**Patients’ suggestions for sexual care**	*N* = 45	%
In your opinion, which method would be useful and/or appropriate to address the sexual problems (several answers possible)?		
Consultation with a caregiver trained in sexology		
Strongly disagree/disagree	2	4.4
No opinion	2	4.4
Agree/fully agree	41	91.2
Consultation with a psychiatrist		
Strongly disagree/disagree	11	24.4
No opinion	16	35.6
Agree/fully agree	18	40.0
Consultation with a sexologist		
Strongly disagree/disagree	6	13.3
No opinion	9	20.0
Agree/fully agree	30	66.7
Couple consultation		
Strongly disagree/disagree	6	13.3
No opinion	7	15.6
Agree/fully agree	32	71.1
Group consultation		
Strongly disagree/disagree	26	57.8
No opinion	8	17.8
Agree/fully agree	11	24.4
On-line therapy using on-line questionnaires and consultations		
Strongly disagree/disagree	14	31.1
No opinion	10	22.2
Agree/fully agree	21	46.7
Pelvic floor physical therapy		
Strongly disagree/disagree	2	4.4
No opinion	10	22.2
Agree/fully agree	33	73.4
Vaginal moisturizer		
Strongly disagree/disagree	1	2.2
No opinion	9	20.0
Agree/fully agree	35	77.8

*N*, number of women who answered the question

Most patients (73.3%) had not received or did not remember receiving any information about sexuality from their oncologist or any other caregiver. Among the 12 patients who had received information, 10 (83.3%) were satisfied with the received information. Most patients (86.7%) had never discussed potential sexual problems with their oncologist or caregiver.

When asked why they did not discuss about sex with them, most patients declared having no question about sexuality (69.2%, agree or fully agree) and feeling it was a minor issue as compared to cancer and the cancer treatment (66.7%). Only a minority of patients felt that the caregivers were too busy to discuss (25.7%) or felt discomfort (30.8%) to talk about the subject.

At the same time, most patients (88.9%) declared it important that sexuality and its potential problems should be discussed with caregivers and that the partner should be involved in the discussion (73.4%). Half of the patients (53.3%) wished for a systematic initial consultation with a sexologist, and 75.6% wished that such a consultation would be available when needed.

The majority of patients (60%) had tried at least one method to overcome their sexual problems, in order of frequency: local treatment like vaginal moisturizer (48.9%), consultation with a psychologist (17.8%), while a consultation with a sexologist remained rare (2.2%). Most of these interventions (63%) originated from the patient herself. Eight patients declared having used other help: consultation with a gynecologist (*n* = 5), with a general practitioner (*n* = 2), and with a midwife (*n* = 1).

Patients suggested as desirable the following interventions in order of frequency: consultation with a caregiver trained in sexology (91.1%), vaginal moisturizer (77.8%), pelvic floor physical therapy (73.4%), combined consultation with her partner and a caregiver (71.1%), consultation with a sexologist (66.7%), online psychotherapy (46.7%), psychiatric consultation (40%), and group consultation (24.4%).

## Discussion

This study described the sexual and global QoL of 45 young BC women during adjuvant endocrine treatment for at least 24 months, as well as the actions taken and suggested by these women to overcome the encountered problems.

First of all, women displayed a high level of interest in the study, as demonstrated by the 83% participation rate and a high rate of response to the items of the questionnaire.

The scores observed for the sexual QoL evaluation with the EORTC SHQ-22 were similar to those previously observed using the same questionnaire in a population without age restriction [9] during endocrine therapy (sexual satisfaction scores were 43.43 and 45.80 in the previous and current studies, respectively), with, though, a higher importance for sexual activity in this younger population (scores of 42.95 and 60.00, respectively). The observed score deteriorations were similar in both studies, with an extremely low score for communication with professionals and a high level of alteration of the sexual satisfaction and libido scores. This deleterious effect of BC and its treatment on sexual QoL is consistent with what has been shown in the literature with other questionnaires [17–21] and is significant when compared to the French general population, in which the rate of sexual satisfaction of women was 88% in a 2006 survey including 6824 women [22]. It should be noted that the professional activity, physical activity, and body mass index rates of our study population were similar to those of the same-aged general population [23, 24].

The global QoL deterioration analyzed with the QLQ-C30 and BR23 was also similar to what we previously reported [9], with the most frequent symptoms being fatigue and insomnia.

We explored the actions taken and proposed by patients to overcome these sexual dysfunctions using a homemade questionnaire specific to our study. Firstly, most patients had not received any information; either a fortiori help from their oncologist nor any caregiver. A vast majority of them declared that is was important that sexuality should be discussed and that their partner should be involved. However, among the women who had received information from their oncologist or any caregiver, the vast majority were satisfied with the received information, which is altogether encouraging, since these caregivers were not trained before the study.

The majority of patients used interventions to overcome sexual problems on their own initiative, underlying the need for specific management of this issue. They stated, mostly not based on their personal experience, that it would be desirable to develop, by order of frequency: consultation with a caregiver trained in sexology, vaginal moisturizer, pelvic floor physical therapy, consultation with their partner, consultation with a sexologist, and less frequently, any kind of psychotherapy.

Most patients declared that sexuality remains a minor issue compared to cancer treatment as the main reason to explain why they did not discuss it with their oncologist or caregiver. This seemingly contradictory response might reflect the patients’ ambivalence about the theme and/or the fact that they want to prioritize the time with their oncologist to focus on the medical information regarding the cancer and its treatment. The role of the oncologist is probably mostly to raise the subject with the patient and question the need for such a dedicated consultation.

The main limitations of our study are the low number of patients included and the absence of longitudinal evaluation. Moreover, a memory bias might have altered the content of the reported events when patients were asked about their history of sexual problems. The strengths are the homogeneity of this young women’s population as well as the use of standardized EORTC questionnaires integrating physical, psychological, and social aspects to evaluate sexual QoL. Moreover, despite the fact that the specific Cupidon questionnaire is not a validated research instrument, asking women their opinion on the actions that could be useful and appropriate to develop in clinic is crucial. This latter information from the involved women going through this issue is precious and should be used to identify the actions to be taken to support these women. The key proposals from the affected patients should be considered to tailor the patient’s management, with the final goal being to improve the QoL of our patients with adequate intervention and support.

## Supplementary Information

Below is the link to the electronic supplementary material.

Supplementary file1 (DOCX 25 KB)

## Data Availability

The data that support the findings of this study are available from the authors upon reasonable request.

## Code availability

Statistical code can be made available on written request to the corresponding author.
