# Supplementary material for: Sexual quality of life assessment in young women with breast cancer during adjuvant endocrine therapy and patient-reported supportive measures
Source: Support Care Cancer. 2022 Jan 14;30(4):3633–41. doi: 10.1007/s00520-022-06810-3 (PMC8857103; doi:10.1007/s00520-022-06810-3)
Supplement: Supplementary file 1 — Supplementary file1 (DOCX 25 KB) [file 520_2022_6810_MOESM1_ESM.docx]

Supplemental data :

**The Cupidon questionnaire**

Date: **|__**|**__|__**|**__|__**|**__**|**__**|**__|**

1. **Did you receive any information from the medical oncologist or another caregiver on the possible impacts of cancer and of your treatment on sexuality?**

- Yes
- No
- I don’t remember

1. **I’m satisfied with the information I received about the possible impacts of cancer and the treatments on my sexuality**

- Strongly disagree
- Disagree
- No opinion
- Agree
- Fully agree

1. **Have you ever had any discussion with the oncologist or another caregiver from the Cancer Center about potential sexual problems?**

- Yes
- No
- I don’t remember

1. **I did not discuss about sexuality because:**

|  | **Strongly disagree** | **Disagree** | **No opinion** | **Agree** | **Fully agree** |
| --- | --- | --- | --- | --- | --- |
| 1. I had no question or I didn’t feel the need to discuss about it |  |  |  |  |  |
| 1. I considered sexuality as a minor issue compared to cancer treatment |  |  |  |  |  |
| 1. Physician and/or nurses looked in a hurry or too busy |  |  |  |  |  |
| 1. I felt too shy and/or discomfort to discuss this topic |  |  |  |  |  |
| 1. I felt discomfort since my physician was a man and/or since I had no positive contact with him/her |  |  |  |  |  |
| 1. It is in contradiction with my education, beliefs, culture |  |  |  |  |  |

1. **In my opinion, it is important that a physician and/or a caregiver from the Cancer Center addresses the issue of sexuality and its potential dysfunction due to the cancer or to cancer treatments:**

- Strongly disagree
- Disagree
- No opinion
- Agree
- Fully agree

1. **In my opinion, it is important that a physician and/or a caregiver from the Cancer Center addresses the issue of sexuality and its potential dysfunction due to the cancer or to cancer treatments with my partner:**

- Strongly disagree
- Disagree
- No opinion
- Agree
- Fully agree
- Not applicable (no partner)

1. **I would have wished that a consultation about sexuality was systematically proposed at the beginning of cancer treatment:**

- Strongly disagree
- Disagree
- No opinion
- Agree
- Fully agree
- Not applicable (no partner)

1. **I would have wished that a consultation about sexuality was available in the Cancer Center when needed during my treatment:**

- Strongly disagree
- Disagree
- No opinion
- Agree
- Fully agree
- Not applicable (no partner)

1. **Have you been using any of these methods for sexual purpose since the beginning of your disease?**

- Consultation with a psychologist: 🞎 Yes 🞎 No
- Consultation with a psychiatrist: 🞎 Yes 🞎 No
- Consultation with a sexologist: 🞎 Yes 🞎 No
- Pelvic floor physical therapy: 🞎 Yes 🞎 No
- Use of vaginal moisturizer: 🞎 Yes 🞎 No
- Use of vaginal laser therapy: 🞎 Yes 🞎 No
- Other : ……………………………………………………………………………………………………

……………………………………………………………………………

1. **If you have been using any method from the previous question, have you decided it on your own?**

- Yes
- No, with the advice of my oncologist
- No, with the advice of my general practician
- Other

1. **In your opinion, which method would be useful and/or appropriate to address the sexual problems (several answers possible)?**

|  | **Strongly disagree** | **Disagree** | **No opinion** | **Agree** | **Fully agree** |
| --- | --- | --- | --- | --- | --- |
| 1. Consultation with a caregiver trained in sexology |  |  |  |  |  |
| 1. Consultation with a psychiatrist |  |  |  |  |  |
| 1. Consultation with a sexologist |  |  |  |  |  |
| 1. Couple consultation |  |  |  |  |  |
| 1. Group consultation |  |  |  |  |  |
| 1. On-line therapy using on-line questionnaires and consultations |  |  |  |  |  |
| 1. Pelvic floor physical therapy |  |  |  |  |  |
| 1. Vaginal moisturizer |  |  |  |  |  |
| 1. Other |  | | | | |
